# Does body mass index influence surgical options and overall survival in breast cancer patients?

**DOI:** 10.1007/s12094-023-03154-0

**Published:** 2023-04-04

**Authors:** Carla Luís, Rute Fernandes, João Dias, Deolinda Pereira, Firmino Machado, Pilar Baylina, Rúben Fernandes, Raquel Soares

**Affiliations:** 1 Biochemistry Unit, Department of Biomedicine, Faculty of Medicine, University of Porto (FMUP), Al Prof Hernâni Monteiro, 4200-319 Porto, Portugal; 2 i3S - Instituto de Investigação e Inovação em Saúde, Universidade do Porto, Porto, Portugal; 3 Portuguese Oncology Institute of Porto (IPO-Porto), Porto, Portugal; 4 EPIUnit–Instituto de Saúde Pública, University of Porto, Porto, Portugal; 5 Public Health Unit, ACES Porto Ocidental, Alto Ave Hospital Center, Guimarães, Portugal; 6 School of Health, Polytechnic of Porto (ESS/P.PORTO), Porto, Portugal; 7 Faculty of Health Sciences, University Fernando Pessoa, Fernando Pessoa Hospital-School (FCS/HEFP/UFP), Porto, Portugal

**Keywords:** Breast cancer, Obesity, Surgery, Mastectomy, Sentinel node biopsy, Axillary dissection

## Abstract

Obesity is a relevant risk factor in breast cancer (BC), but little is known about the effects of overweight and obesity in surgical outcomes of BC patients. The aim of this study is to analyse surgical options and associated overall survival (OS) in overweight and obese women with BC. In this study, 2143 women diagnosed between 2012 and 2016 at the Portuguese Oncology Institute of Porto (IPO-Porto) were included, and the clinicopathological information was retrieved from the institutional database. Patients were stratified by body mass index (BMI). Statistical analysis included Pearson's chi-squared test with statistical significance set at *p* < 0.05. Multinomial, binary logistic regression and cox proportional-hazards model were also performed to calculate odd ratios and hazard ratios with 95% confidence intervals for adjusted and non-adjusted models. The results revealed no statistical difference in histological type, topographic localization, tumour stage and receptor status and in the number of surgical interventions. Overweight women have increased probability to be subjected to sentinel node biopsy. Obese and overweight women are more likely to be submitted to conservative surgery and contrariwise, less likely to undergo total mastectomy. Patients submitted to conservative surgery and not submitted to total mastectomy had a favourable OS although without statistical significance. No significant differences were observed in OS when stratified by BMI. Our results revealed significant variations regarding the surgical options in overweight and obese patients, but these were not translated in OS difference. More research is recommended to better address treatment options in overweight and obese BC patients.

## Introduction

Breast cancer (BC) is a complex heterogeneous pathology with high incidence and mortality rates amongst women. Therapeutical strategies are associated not only with tumour biology (like molecular subtype, differentiation grade, histological type, lymphovascular invasion, etc.) but also with the extent of the disease, breast size, tumour stage, and patient preferences [1]. Treatment options includes chemo and radiotherapy, endocrine therapy with or without targeted biomarkers, however, surgery remains the cornerstone of BC treatment and is, in most cases, the number one option [1]. The evolution of BC surgery has evolved from radical mastectomy as the only surgical treatment to a more comprehensive approach with several alternative conservative methods, which comprises an aesthetic basis [2].

Mastectomies are the upmost used procedures, conservative surgery (CS), also designated partial mastectomy, consists in the removal of the malignant tissue and surrounding margins with satisfactory results regarding long-term survival and aesthetical effect [3]. CS is considered a safe procedure, associated with low rates of local recurrence and distant metastasis, low positive margins rate and low surgical complications [4]. The total mastectomy (TM) implies the excision of all breast tissue, skin, nipple, and areola whilst radical mastectomy (RM) includes the same procedures with additional excision of chest wall muscles and lymph node dissection. Previous results showed no survival advantage of radical mastectomy over total mastectomy with or without radiation therapy in a clinical follow-up of 25 years [5]. Another surgical alternative is mastectomy with immediate reconstruction (MR), which is associated with considerable improvement on aesthetic outcomes [6]. Surgical approaches also include sentinel node biopsy (SNB) and axillary dissection (AD). A previous study focussing on patients with BC with N1 positive sentinel nodes found no significant differences in overall and free specific survival in patients submitted to adjuvant axillary radiotherapy as compared to AD. SNB with adjuvant therapy seems to promote secure regional control [7], without the AD-associated co-morbidities [8].

Discrepancies associated with psychosocial factors [11], racial differences [9, 10], and body mass index (BMI) [11] are described as potential modulators in the selection and response to the surgical procedure. Numerous results have revealed that obesity is a depraved risk factor in BC [12]. Regarding surgical outcomes, it was already reported that obese women have more surgical complications and are less likely to be submitted to breast reconstruction [11]. Also, obese women are associated with decreased sentinel node identification due to failure in map rates [11]. To better addressed the association between surgery strategies in obese and overweight women, we performed a retrospective study associating surgery metrics and overall survival (OS) to uncover potential associations.

## Materials and methods

### Study population and ethical approval

This study was conducted according to the national and international ethical recommendations and approved by the Ethics Committee of the Portuguese Institute of Oncology of Porto FG, EPE (IPO-Porto). Clinicopathological and demographic information from women diagnosed in IPO-Porto between 2012 and 2016 were extracted from the institutional database of Outcomes Research Lab. Collected data was compiled in a database titled “Deciphering Obesity and Cancer” (DOC). For this study, women without surgical information were excluded from this study.

### Methods

BMI was calculated using the Quetelet index measured up to 120 days after diagnosis. BMI was grouped in 3 categories: normal (BMI between 18.5 and 25 kg/m^2^), overweight (BMI between 25 and 30 kg/m^2^), and obese (BMI above 30 kg/m^2^) [13]. Histological types were grouped in “invasive ductal carcinoma” (IDC), which included invasive ductal carcinoma with other types of carcinomas, “invasive lobular carcinoma” (ILC), which included invasive lobular carcinoma with other types of carcinomas and “other histological types”. Other types of carcinomas refers to the classification of tumours of the breast by the World Health Organization (WHO) and include Apocrine adenoma, Metaplastic carcinoma, Invasive micropapillary carcinoma, amongst others [14]. Hormonal receptors were classified as positive or negative, cut-off of 1% of tumour positive cells. HER2 status was assessed by immunohistochemistry and validated by fluorescence in situ hybridization (FISH) according to the American Society of Clinical Oncology/College of American Pathologists (ASCO/CAP) guidelines [15]. Immunohistochemistry for receptor status was performed at the pathology department according to standard protocols. Tumour stage was assessed according to the 7th edition of the American Joint Committee on cancer by tumour node metastasis (TNM) system [16]. For topographic localization, cases were divided in the four quadrants—inferior outer quadrant (IOQ), inferior inner quadrant (IIQ), superior outer quadrant (SOQ), superior inner quadrant (SIQ), other localizations including nipple, skin, central quadrant, and axillary extension (Other) and overlapping lesions (Multiple). OS was measured in months, from the date of diagnosis till death date or date of the study end (16 December 2021). The surgery analysis included 6 types of surgical intervention: Sentinel Node Biopsy (SNB), Axillary Dissection (AD), Conservative Surgery (CS), Total Mastectomy (TM), Radical Mastectomy (RM), and Mastectomy and Reconstruction (MR). Quantification of the number of surgeries consisted in the sum of surgical procedures, independently of the date.

### Statistical analysis

Statistical analysis was performed using IBM^®^ SPSS^®^, version 27 software. Descriptive statistics was used for data description. Age at diagnosis as a continuous variable was presented as mean ± standard deviation and range, tested for normality with Shapiro–Wilk test and analysed by one-way Anova. The first approach consisted in a Pearson's chi-squared test to analyse the association between BMI and variables. Significance was set at *p* < 0.05. Binary, multinomial logistic regression and cox proportional-hazards model were used to compute odd ratios (OR) and hazard ratios (HR) with 95% confidence intervals (CI) in a non-adjusted model and adjusted for age at diagnosis, histological type, stage at diagnosis, and receptor status. Cox proportional-hazard analysis were based in the follow-up time from date of diagnosis until date of event or censoring, death, or end of follow-up (truncated at 10 years). Additional analysis was performed to CS and TM to calculate the level of significance for surgical options and stratified by BMI.

## Results

From the 2143 women included, 734 (34.2%) were normal-weighted, 807 (37.7%) were overweighted and 602 (28.1%) exhibited obesity. Table 1 displays the clinicopathological and surgical information, with statistical analysis of BC patients stratified by BMI categories. A statistically significant association was found between BMI and conservative surgery, and BMI and total mastectomy. The results of clinicopathological parameters revealed no statistical significance between BMI and tumour features like histological type, receptor status, tumour stage and topographic localization. We also observed a positive correlation between BMI and age at diagnosis.

**Table 1:** Clinicopathological and surgical information with statistical analysis of breast cancer patients stratified by body mass index categories

	Normal-weight (BMI 18.5–24.9)	Overweight (BMI 25–29.9)	Obese (BMI > 30)	Total	*p* value
	*N*	*n*	*n*	*N*	
BMI distribution	734 (34.2%)	807 (37.7%)	602 (28.1%)	2143 (100%)	–
Age					
Mean ± SD	51.8 ± 11.9	56.9 ± 11.0	58.8 ± 9.9	55.7 ± 11.4	< 0.001*
Range	24–87	27–88	33–86	24–88	
Histological type					
IDC	593 (80.8%)	670 (83.0%)	477 (79.2%)	1740 (81.2%)	0.076
ILC	59 (8.0%)	71 (8.8%)	48 (8.0%)	178 (8.3%)	
Other	82 (11.2%)	66 (8.2%)	77 (12.8%)	225 (10.5%)	
Receptor status					
ER + /PR +	426 (58.0%)	482 (59.7%)	388 (64.5%)	1296 (60.5%)	0.056
ER + /PR-	76 (10.4%)	63 (7.8%)	42 (7.0%)	181 (8.4%)	
HER2 +	138 (18.8%)	164 (20.3%)	93 (15.4%)	395 (18.5%)	
Triple negative	84 (11.4%)	85 (10.5%)	65 (10.8%)	234 (10.9%)	
Missing data	10 (1.4%)	13 (1.6%)	14 (2.3%)	37 (1.7%)	
Pathological tumour stage					
Stage I	320 (43.6%)	340 (42.1%)	226 (37.5%)	886 (41.4%)	0.137
Stage II	274 (37.3%)	311 (38.6%)	236 (39.2%)	821 (38.3%)	
Stage III	122 (16.6%)	144 (17.9%)	127 (21.1%)	393 (18.3%)	
Stage IV	18 (2.5%)	11 (1.4%)	13 (2.2%)	42 (2.0%)	
Missing data	0 (0.0%)	1 (0.0%)	0 (0.0%)	0 (0.0%)	
Topographic localization					
SIQ	66 (9.0%)	81 (10.0%)	53 (8.8%)	200 (9.3%)	0.709
SOQ	223 (30.4%)	251 (31.1%)	177 (29.4%)	651 (30.4%)	
IIQ	35 (4.8%)	34 (4.2%)	29 (4.8%)	98 (4.6%)	
IOQ	53 (7.2%)	37 (4.6%)	36 (6.0%)	126 (5.9%)	
Other	35 (4.8%)	48 (6.0%)	34 (5.7%)	117 (5.5%)	
Multiple	322 (43.8%)	356 (44.1%)	273 (45.3%)	951 (44.3%)	
Number of surgeries					
1	122 (16.6%)	126 (15.6%)	107 (17.8%)	355 (16.6%)	0.899
2	450 (61.3%)	504 (62.5%)	362 (60.1%)	1316 (61.4%)	
3	130 (17.7%)	147 (18.2%)	114 (18.9%)	391 (18.2%)	
4	28 (3.8%)	27 (3.3%)	18 (3.0%)	73 (3.4%)	
5	4 (0.6%)	3 (0.4%)	1 (0.2%)	8 (0.4%)	
Surgical procedure					
SNB	513 (67.1%)	585 (70.4%)	411 (67.5%)	1509 (68.4%)	0.213
AD	163 (21.3%)	182 (21.9%)	144 (23.6%)	489 (22.2%)	0.740
CS	337 (44.1%)	448 (53.9%)	332 (54.5%)	1117 (50.7%)	< 0.001*
TM	293 (38.3%)	257 (30.9%)	178 (29.2%)	728 (33.0%)	< 0.001*
RM	156 (20.4%)	154 (18.5%)	132 (21.7%)	442 (20.0%)	0.373
MR	6 (0.8%)	1 (0.1%)	1 (0.2%)	8 (0.4%)	0.051

*BMI*, Body Mass Index; *SD*, Standard deviation; *SNB*, Sentinel Node Biopsy; *AD*, Axillary Dissection: *CS*, Conservative Surgery; *TM*, Total Mastectomy; *RM*, Radical Mastectomy; *MR* – Mastectomy and Reconstruction

*Statistical significance highlighted in bold (*p* value < 0.05)

### Number of surgical interventions

We did not observe any associations between BMI and the number of surgical procedures per patient (Table 2). There was no statistical evidence that overweight and obese patients are submitted to more surgical procedures. We found some inconsistencies between the number of surgeries and number of total surgical procedures. We observed that some types of surgical approaches were performed more than once on the same patient and variable surgical procedures is presented as number of patients submitted to that specific surgery. Reconstructive procedures or reinterventions due to surgical complications were not included in the number of surgeries.

**Table 2:** Multinomial logistic regression for number of surgeries

*N*^#^		*P* value	OR	95% CI		*P* value	OR	95% CI	
				Lower	Upper			Lower	Upper
2	Normal-weight	–	1	–	–	–	1	–	–
	Overweight	0.570	1.084	0.820	1.434	0.414	1.152	0.820	1.620
	Obese	0.565	0.917	0.683	1.231	0.515	1.129	0.784	1.627
3	Normal-weight	–	1	–	–	–	1	–	–
	Overweight	0.605	1.095	0.777	1.543	0.265	1.240	0.850	1.808
	Obese	0.999	1.000	0.697	1.435	0.260	1.262	0.842	1.891
4	Normal-weight	–	1	–	–	–	1	–	–
	Overweight	0.818	0.934	0.521	1.675	0.665	1.150	0.612	2.161
	Obese	0.346	0.733	0.384	1.399	0.665	1.167	0.580	2.346
5	Normal-weight	–	1	–	–	–	1	–	–
	Overweight	0.679	0.726	0.159	3.312	0.895	1.113	0.227	5.465
	Obese	0.265	0.285	0.031	2.590	0.619	0.555	0.054	5.651

*N*, number of surgeries; *OR*, odd ratio; *CI*, confidence interval

^#^Reference category: 1 surgical intervention

^§^Adjusted to age at diagnosis, histological type, tumour stage and receptor status

### Type of surgical procedures

Regarding surgical procedures, the statistical evaluation did not achieve any significant differences in AD, RM and MR. Overweight women have increased OR to undergo SNB (*p* value 0.038; OR 1.317; 95% CI 1.016–1.707). Not only overweight, but also obese women are more likely to be submitted to CS and less likely to undergo total mastectomy (Table 3). Obese patients have twice the risk to be submitted to CS (*p* value  < 0.001; OR 2.059; 95% CI 1.608–2.637) and half the risk to be submitted to TM (*p* value  < 0.001; OR 0.569; 95% CI 0.447–0.725). Overweight women with BC have a 78% increased risk to be submitted to CS (*p* value  < 0.001; OR 1.780; 95% CI 1.422–2.228) and a 35.2% decreased risk to be submitted to TM (*p* value  < 0.001; OR 0.648; 95% CI 0.521–0.805).

**Table 3:** Crude and adjusted binary logistic regression for each surgical procedure

	Crude					Adjusted^§^			
	*P* value	OR		95% CI		*P* value	OR	95% CI	
				Lower	Upper			Lower	Upper
Sentinel Node Biopsy									
BMI Categories	0.213		–	–	–	0.114	–	–	–
Normal-weight	–		1	–	–	–	1	–	–
Overweight	0.260		1.135	0.910	1.416	0.038*	**1.317**	**1.016**	**1.707**
Obese	0.524		0.927	0.734	1.170	0.244	1.180	0.893	1.560
Axillary dissection									
BMI Categories	0.740		–	–	–	0.991	–	–	–
Normal-weight	–		1	–	–	–	1	–	–
Overweight	0.871		1.020	0.803	1.297	0.932	0.989	0.769	1.271
Obese	0.459		1.101	0.853	1.422	0.967	1.006	0.766	1.321
Conservative surgery									
BMI Categories	< 0.001*		**–**	**–**	**–**	< 0.001*	**–**	**–**	**–**
Normal-weight	–		1	–	–	–	1	–	–
Overweight	< 0.001*		**1.470**	**1.203**	**1.797**	< 0.001*	**1.780**	**1.422**	**2.228**
Obese	< 0.001*		**1.449**	**1.167**	**1.799**	< 0.001*	**2.059**	**1.608**	**2.637**
Total Mastectomy									
BMI Categories	< 0.001*		**–**	**–**	**–**	< 0.001*	**–**	**–**	**–**
Normal-weight	–		1	–	–	–	1	–	–
Overweight	< 0.001*		**0.703**	**0.571**	**0.867**	< 0.001*	**0.648**	**0.521**	**0.805**
Obese	< 0.001*		**0.632**	**0.503**	**0.795**	< 0.001*	**0.569**	**0.447**	**0.725**
Radical Mastectomy									
BMI Categories	0.373		–	–	–	0.486	–	–	–
Normal-weight	–		1	–	–	–	1	–	–
Overweight	0.289		0.874	0.681	1.121	0.248	0.834	0.613	1.135
Obese	0.766		1.041	0.801	1.352	0.389	0.864	0.620	1.205
Mastectomy and reconstruction									
BMI Categories	0.100		–	–	–	0.319	–	–	–
Normal-weight	–		1	–	–	–	1	–	–
Overweight	0.080		0.151	0.018	1.253	0.164	0.217	0.025	1.867
Obese	0.139		0.202	0.024	1.682	0.403	0.387	0.042	3.584

*OR*, Odd ratio; *CI*, Confidence interval

*Statistical significance highlighted in bold (*p* value < 0.05)

^§^Adjusted to age at diagnosis, histological type, tumour stage and receptor status

### Overall survival

To further access the impact of surgical procedures in OS, we performed a Cox proportional-hazard model stratified by BMI exclusively and by CS and TM. No significant differences were achieved and when compared to the OS reference, no significant discrepancies were observed (Table 4).

**Table 4:** Crude and adjusted Cox proportional-hazard model for overall survival

	Crude				Adjusted^§^			
	*P* value	HR	95% CI		*P* value	HR	95% CI	
			Lower	Upper			Lower	Upper
BMI (reference)								
BMI	0.270	–	–	–	0.360	–	–	–
Normal	–	1	–	–	–	1	–	–
Overweight	0.264	1.186	0.880	1.598	0.282	1.183	0.871	1.605
Obese	0.115	1.303	0.937	1.811	0.173	1.270	0.901	1.790
Conservative surgery								
BMI	0.228	–	–	–	0.296	–	–	–
Normal	–	1	–	–	–	1	–	–
Overweight	0.224	1.204	0.893	1.625	0.235	1.204	0.886	1.637
Obese	0.095	1.325	0.952	1.843	0.137	1.300	0.920	1.837
Total Mastectomy								
BMI	0.264	–	–	–	0.329	–	–	–
Normal	–	1	–	–	–	1	–	–
Overweight	0.254	1.190	0.882	1.606	0.257	1.194	0.879	1.622
Obese	0.112	1.307	0.939	1.819	0.156	1.283	0.909	1.812

*HR*, Hazard Ratio; *CI*, confidence interval

^§^Adjusted to age at diagnosis, histological type, tumour stage and receptor status.

**Fig. 1:**
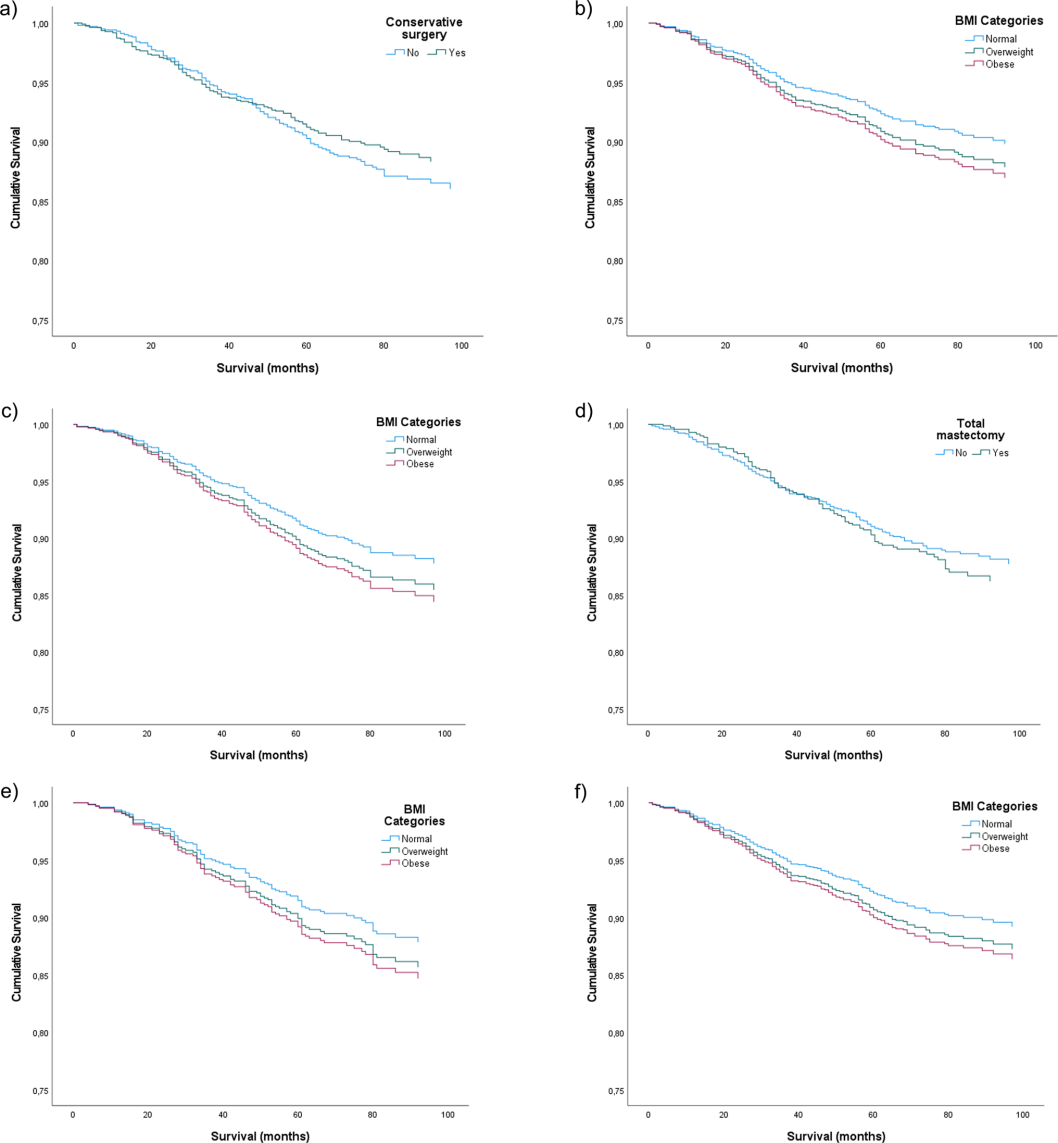
Survival plot of Cox proportional-hazard model adjusted to age at diagnosis, histological type, tumour stage and receptor status. **a** Overall survival (OS) of conservative surgery intervention (*p* value = 0.296); **b** OS of conservative surgery intervention stratified by BMI (*p* value  = 0.853); **c** OS of no conservative surgery stratified by BMI (*p* value  = 0.346); **d** OS of total mastectomy intervention (*p* value  = 0.329); **e** OS of total mastectomy intervention stratified by BMI (*p* value  = 0.608); **f** OS of no total mastectomy intervention stratified by BMI (*p* value = 0.595)

Whilst Table 3 displays the results from analysis using ‘surgery’ as *strata*, Fig. 1 presents the results from independent statistical analysis for each condition. No statistical significance was achieved. We observed that survival curve favours women who were submitted to conservative surgery (Fig. 1a) and were not submitted to total mastectomy (Fig. 1d). BMI stratification presented a similar pattern with obese women presenting a lower OS (Fig. 1b, c, e, f).

## Discussion and conclusions

Obesity is an important risk factor in tumourigenesis and tumour progression for several types of cancer including BC [17]. Obese BC metabolism is associated with many different mechanisms, since adipose tissue releases several modulatory factors like adipokines, cytokines, inflammatory mediators, free fatty acids, hypoxia inducible factors, and others that activate, promote, and mediate relevant metabolic pathways closely involved in tumour biology [17].

The complex connection between both pathologies results in harshest outcomes namely more aggressive cancers, decreased overall and disease-free survival, increased and severed postoperative complications [11]. Studies reported that women with obesity have increased risk to more extensive axillary dissection interventions [18], are less likely to undergo mastectomy [19] and reconstruction after mastectomy [12]. Moreover, patients with BC and obesity have extended clinical hospitalization [19], and serious surgery-related complications, such as infections, haemorrhages, wound dehiscence, prosthetic and flap loss, venous thromboembolism, lymphoedema and pneumonia [20].

The focus of our work was to evaluate the surgical options and outcomes of patients with normal weight, overweight and with obesity. Our results disclosure that overweight and obese female patients are more likely to be submitted to conservative surgery and less likely to undergo mastectomy. Moreover, women with overweight are more likely to be submitted to sentinel node biopsy.

Conservative surgery is usually prescribed in early tumour stages and in cases presenting low tumour-breast volume ratio. Also, it is recommended that CS should be planned to include an immediate reconstruction. The total mastectomy is reserved for patients with more aggressive tumours, invasive advanced carcinomas and when breast-tumour volume ratio does not allow to conserve the breast. The finding that overweight and obese women are more likely to undergo CS led us to hypothesize that the accumulation of adipose tissue observed in overweight and obese women lead to a low tumour-breast volume ratio. CS is also prescribed in early tumour stages. However, no significant differences in tumour stage were observed, and additionally, advanced tumour stages have a lower incidence rate in the obese category when compared to normal weight category. Regarding the results for SNB, the previous studies concluded that women with obesity present higher recurrence incidence and axillary dissection failure rates [18]. However, we did not find statistically significance in obese patients, but rather in overweight women. SNB represents one of the forms of axillary lymph node involvement evaluation that is mandatory for staging (N). The evaluation can be clinical (whenever suspicious nodes are present on the physical examination) or pathological evaluation (pN), which is the parameter considered in the staging (pTNM). Women undergoing TM (with lymph node emptying) are not submitted to SNB If the disease is more invasive, it will ultimately lead to AD. It is well documented that overweight shares common outcomes with obesity [21]. Another interesting observation is the negative result in radical mastectomy. Although without significance, overweight and obese BC patients are less likely to be submitted to radical mastectomy. Considering the obesity-increased tumour aggressiveness described in the literature, an increased OR between women with obesity and radical mastectomy would be expected.

We believe that our results enclosure new information regarding treatment choices in women with obesity, although we are aware of the study limitations. OS is a variable that comprises multiple influencing factors. On one hand, different adjuvant therapy strategies, which was not included because it was beyond the scope of our work. On the other hand, the presence of obesity-associated pathologies such as diabetes or cardiovascular complications. We also considered that the available data regarding disease-free-survival could be biased due to the constrictions derived from the pandemic condition. Moreover, we believe that the menopausal status is an important trait to be addressed, but we could not access this information. Furthermore, surgical interventions may also result from some subjective factors like patient's willing and doctors' preferences, which are impractical to include in the statistical analysis.

We concluded that yes, obesity influences surgical options, but not outcomes. We agree with the authors who state that obesity should be addressed in BC, since it does not only modulate tumour characteristics, but also unfolds surgical interventions. Some authors also defend the inclusion of bariatric surgery in disease management. It was already documented that BC risk decreases after bariatric surgery [22] since bariatric surgery outcomes include alterations in the breast tissue composition metrics [23] by lowering the breast density, which is another BC risk factor [24]. Finally, we would like to stress that the implementation of targeted guidelines for patients with obesity is of upmost importance to improve recovery rates and life quality.

## Data Availability

The datasets generated and/or analysed during the current study are not publicly available due to privacy or ethical restrictions.
